# “I felt like she didn’t take me seriously”: a multi-methods study examining patient satisfaction and experiences with polycystic ovary syndrome (PCOS) in Canada

**DOI:** 10.1186/s12905-022-01630-3

**Published:** 2022-02-23

**Authors:** Miya Ismayilova, Sanni Yaya

**Affiliations:** 1 Interdisciplinary School of Health Sciences, University of Ottawa, Ottawa, Canada; 2 School of International Development and Global Studies, Faculty of Social Sciences, University of Ottawa, 120 University Private, Ottawa, ON K1N 6N5 Canada; 3 The George Institute for Global Health, Imperial College London, London, UK

**Keywords:** PCOS, Polycystic ovary syndrome, Survey, Interviews, Satisfaction, Experiences, Diagnosis, Barriers, Facilitators

## Abstract

**Background:**

Polycystic ovary syndrome (PCOS) is a common and complex condition affecting metabolic, reproductive, cardiovascular, and psychological health in women. Previous studies point to widespread dissatisfaction and delays with the diagnosis of PCOS and a lack of information provision by doctors, with few studies on Canadian populations. This multi-methods study explored the perceptions and experiences of PCOS diagnosis in Canada using an online-distributed survey and follow-up, in-depth, semi-structured phone interviews.

**Methods:**

The online questionnaire was completed by 296 women aged 18–60 with a self-reported diagnosis of PCOS. The survey measured time to diagnosis, number of doctors seen, and information provided. Descriptive statistics, Chi-square tests, Fisher’s exact tests, and Spearman’s rank correlations were used to analyze the sample characteristics and correlations between the demographic factors and the outcome measures. Twenty-five follow-up interviews were held over the phone and analyzed using thematic analysis and interpretive description methodology.

**Results:**

Survey respondents were dissatisfied with the information provided about PCOS (66%), lifestyle management (34%), and medical therapy (38%) at the time of diagnosis. Approximately 34% of respondents waited for more than 2 years and 41% saw 3 or more doctors before attaining diagnosis. Many did not receive any information about lifestyle management (42%) or medical therapy (28%). Interview participants encountered doctors who lacked sufficient knowledge on PCOS to diagnose, chronically dismissed concerns, and did not provide necessary medical information about the condition. Women described benefitting from self-advocation to receive the care they needed from doctors, and self-educating about PCOS using materials they could find online. Younger interview participants whose symptoms began in adolescence would often not understand the significance of symptoms until much later in life, contributing to delayed diagnoses.

**Conclusions:**

Greater education on PCOS for physicians, particularly in primary care, is needed to prevent delayed diagnoses and ensure that patients are provided with reliable medical information about their condition. Greater awareness of PCOS may be necessary for the general population to help women identify symptoms, especially for adolescents and their parental figures.

**Supplementary Information:**

The online version contains supplementary material available at 10.1186/s12905-022-01630-3.

## Background

Polycystic ovary syndrome (PCOS) is a common and complex disorder that affects 6%-10% of women of reproductive age [1]. PCOS affects many areas of health and well-being across the life span due to its reproductive, metabolic, and psychological impacts [2]. PCOS is the most common cause of anovulatory infertility [3]. In addition to infertility, patients with PCOS have greater rates of obesity [4], type 2 diabetes [5], metabolic syndrome [6, 7], cardiovascular risk factors such as hypertension [8, 9], poor body image [10, 11], eating disorders [12–14], anxiety and depression [15–17].

PCOS is diagnosed most with the Rotterdam criteria when 2 out of 3 of the following features are present with the exclusion of other conditions: polycystic ovaries on ultrasound, biochemical/clinical hyperandrogenism, and oligo-amenorrhea [18]. First-line treatments for PCOS include lifestyle management and oral contraceptive pills [19, 20].

It can be difficult for physicians to diagnose PCOS due to heterogeneity in PCOS presentation [21], complex diagnostic criteria [22], controversial diagnostic criteria for adolescent populations [23, 24], and clinical variability in approaches to the diagnosis [25–27]. Previous studies investigating patients’ experiences of PCOS diagnosis have discovered many cases of delays in diagnosis, patients having to see multiple health professionals, and dissatisfaction with the information provided to them about their condition [28–31]. Delays in diagnosis and unmet informational needs can have long-term consequences, such as an increase in depression and anxiety symptoms [32] and reduced ability to self-manage and improve lifestyle to prevent comorbidity [28, 33]. Despite these potential impacts, there have been few comprehensive studies investigating diagnosis experience in diverse age groups of patients with PCOS, particularly in Canada.

This study aims to investigate patients’ experiences and satisfaction with PCOS diagnosis in Canada, including the information provided and whether their needs at the time of diagnosis had been met, accounting for age considerations. The aim will be explored using a multi-method approach: a survey replicated from Gibson-Helm et al.’s (2017) [28] work and follow-up in-depth telephone interviews to address a need for more qualitative studies of patients’ experiences with the diagnosis of PCOS.

## Methods

### Study design

A multi-methods approach was taken to explore experiences and satisfaction with PCOS diagnosis: a cross-sectional online questionnaire followed up with semi-structured phone interviews. The target population were women over the age of 18, with a self-declared PCOS diagnosis made by a medical professional in Canada, and who have resided in Canada since their diagnosis. The sample was a convenience sample drawn from online PCOS groups on Facebook, Reddit, and various PCOS online forums.

The online questionnaire was hosted on SurveyMonkey, a website for constructing, storing, and analysing online surveys. This questionnaire was adapted from a PCOS questionnaire previously used in published research [28]. The original questionnaire was distributed worldwide and developed with input from a multidisciplinary expert advisory group and piloted with women with PCOS and the study provided a foundation for further research to investigate diagnosis experiences in specific countries [28]. Permission to replicate the survey was granted by the authors and publisher in June 2018. The survey was modified to include eligibility questions, such as participants having been diagnosed in Canada and residing in Canada. The first author tested the survey for functionality before fielding. No pilot testing was performed.

Although survey-based studies can describe many concerns of women with PCOS, qualitative interviews capture first-hand accounts and experiences which can be missed by solely questionnaire-based studies. Qualitative research is well suited to generate data on perceptions of health experience and perceptions of care [34]. To garner more novel results for this study, follow-up in-depth, semi-structured interviews were conducted to identify factors related to PCOS diagnosis that were not included in the survey. Interviews explored women’s perceptions and experiences with the diagnosis and management of PCOS, but only the themes relating to their diagnosis experience are explored in this paper.

## Research setting

Survey respondents and interviewees participated from across Canada. Canada is a country with 38,005,238 people as of July 2020, with the 4 most populous provinces being Ontario, Quebec, British Columbia, and Alberta [35]. The three most populous provinces after Ontario (14,734,01) are Quebec (8,574,571), British Columbia (5,147,712), and Alberta (4,421,876) [35]. Surveys were hosted on SurveyMonkey’s Canadian servers and phone interviews were conducted by the first author in a private residential office in Ottawa, Canada.

### Participants and recruitment

Eligibility criteria included age ≥ 18 years, a self-reported diagnosis of PCOS made in Canada, having resided in Canada since the diagnosis, and an ability to read and speak English. No upper age limit was established to help promote participation from older patients living with PCOS, in the peri- and post-menopausal stages of life. Participants were 296 survey respondents and 25 interviewees. Participants were recruited through posts on PCOS groups on Facebook, Reddit, and online PCOS forums. The survey was advertised entirely online by the first author posting a short paragraph about the purpose of the study along with a recruitment poster and a link to the survey on SurveyMonkey. The PCOS Awareness Association also helped with recruitment by doing a one-time re-post on their Facebook page with the study’s recruitment poster and the survey link. Recruitment took place between April and December of 2018. Social media was used as a recruitment strategy to reach a wider sample from across all Canadian provinces. The advertisement posts directed interested respondents to the participant information statement and the questionnaire on SurveyMonkey. The survey was voluntary and open. Upon completion of the questionnaire, participants had an opportunity to submit their contact information (i.e., name and email) for follow-up interviews. Respondents who indicated interest in being interviewed at the end of the questionnaire were subsequently interviewed remotely, over the phone, on a first come first serve basis until preliminary analysis during data collection suggested thematic consistency across most age groups. Interviews were in-depth and lasted around 1 h (see Additional file 1 for interview guide on diagnosis).

### Data collection

The e-survey includes questions on demographics, PCOS diagnosis experience, information provided at diagnosis, and concerns about PCOS features. No question was compulsory. The questionnaire was modified to include screening questions on eligibility criteria (e.g., area of residence, country of diagnosis) to ensure that participants resided in and were diagnosed in Canada. There was a total of 25 items distributed across 5 pages. All items were skippable, and participants were option to use the “Back” and “Next” buttons to review answers. Adaptive questioning was used for a few items to reduce the number of items to fill out where possible. Multiple entries from the same browser were preventing by using SurveyMonkey’s multiple responses option; however, IP addresses were not stored to ensure anonymity. Participants had the option to enter a draw to win 1 of 3 CAD$25 Amazon e-gift cards on the last survey page.

The semi-structured interview guide was developed based on themes and gaps identified in previous literature, and included questions such as, ‘Could you describe a typical day living with PCOS?’ and “How did you first learn about PCOS?” The first author conducted all interviews over the phone in their private residential office with no one else present. Twenty-five interviews were conducted over the phone between October and December 2018, lasted 25–90 min and were audio-recorded and transcribed verbatim. Interviews were capped at 25 once data saturation was reached and no more participants in the 40 + age group were available for interview within the sample. One interview was held over Skype™ due to the participant’s preference. Pseudonyms are used here and in all written documents.

### Quantitative data analysis

Statistical analysis was performed with IBM SPSS Statistics for Windows, version 25 (IBM Corp., Armonk, N.Y., USA). Responses from the survey, including incomplete responses, were exported, and analyzed by the first author. Answers that were obviously false or incorrectly inputted were removed prior to analysis (e.g. height and weight incorrectly inputted). Categorical data were presented as count and proportions and continuous data as mean ± standard deviation (SD) if data followed a normal distribution, or median and interquartile range if data did not follow a normal distribution. A *p* value of 0.05 was considered statistically significant. Chi-square and Fisher’s Exact tests were performed to test for associations between demographic characteristics and experiences of PCOS diagnosis. Spearman’s rank-order correlations were used to assess correlation within satisfaction ratings if the two variables had a monotonic relationship. Variable distribution in the groups was tested using the Shapiro–Wilk test for normality.

### Qualitative data analysis

We coded all qualitative interview data, including field notes made during and after interviews, and managed all data in NVivo 12 (QSR International Pty Ltd. Version 12, 2018). In accordance with Thorne et al.’s (2004) [34] interpretive description methodology, an inductive analysis technique was used to analyze data. Themes were derived entirely from the data. Thorne’s (2004) [34] interpretive description approach is widely used in nursing research and does not generate new truths or theories but rather describes thematic patterns and commonalities while also accounting for individual variations and provides a product that clinicians can use as a backdrop for clinical decision-making. Braun and Clarke’s (2006) [36] six key stages in the thematic analysis of qualitative data were also followed in this study: (1) Familiarize, (2) Generate initial codes, (3) Search for themes, (4) Review themes, (5) Define themes, and (6) Write up the data analysis. Codes and subsequent sub-categories were generated directly from topics raised in the data. The over-arching code categories (barriers and facilitators) were developed from the research questions.

### Trustworthiness

The quantitative study was reported based on the Checklist for Reporting Results of Internet E-Surveys (CHERRIES) [37] and the qualitative study was reported based on the Consolidated criteria for reporting qualitative research (COREQ) [38] (please see Additional files 2 and 3, respectively). At the time of the study, the first author was an MSc student conducting in-depth interviews for the first time after training in graduate classes and workshops. The first author identifies as a female, and participants were made aware of the reason for the author to be conducting this research, their personal interest in the research topic, and PCOS status, but otherwise no significant relationship existed or was established between the author and participants. To ensure reliability and validity, the first author considered researcher bias, used the strategies of thick description, development of a coding system, checking and agreement on themes and analysis by members of the team, transparency when reporting research (as per COREQ), and demonstrating the author’s interpretive lens throughout the report [34].

### Ethical approval and consent to participate

Ethics approval for this multi-methods study was received from the University of Ottawa Research Ethics Boards (REB) in April 2018. Permission to replicate the questionnaire was granted by the authors and Oxford University Press in June 2018. All participants were voluntarily enrolled in the study with free and informed consent. Informed consent for the survey was obtained from all those agreeing to complete the survey. Participants were informed of the purpose of the study, who the investigators were, that their responses will remain confidential and accessible only to the researchers, the length of time of the survey (5–10 min), and that by clicking “I agree” and starting the survey, they were declaring consent to participate but could withdraw their data at any time. Participants were informed of all this via the survey welcome page which also held a link to the full implied consent form. The survey data was hosted solely on the first author’s SurveyMonkey account on Canadian servers and was password-protected. No personal information was linked to survey results in any way (e.g., contact information to enter the draw). The fully de-identified dataset is kept on password-protected computers.

Interview participants were also informed that once they chose to participate, they could withdraw at any time and/or refuse to answer any questions, without suffering any negative consequences. Permission to audio-record the remote interviews was sought and obtained before data collection. All personal identifiers were removed from transcripts and in quoted texts below. Written or oral informed consent was obtained from all interview participants prior to their participation.

## Results

### Socio-demographic characteristics

Advertisement resulted in 397 women accessing the survey, out of which 296 were included in the analysis (response rate of 75%). The rest were screened out due to ineligibility. Mean age of participants was 29 years and median BMI was in the obese range (33 kg/m^2^; Table 1). The results showed that 16.9% of the sample were of average BMI, 0.7% were underweight (BMI < 18.5), 18.8% were of average BMI (BMI 18.5–24.9), and 63.6% of the sample were obese (BMI ≥ 30.0), of which 39.5% were class I (BMI 30.0–34.9, moderate), 26.4% were class II (BMI 35.0–39.9, severe), and 34.1% were class III (BMI ≥ 40, very severe). A substantial proportion of the participants was from Ontario (58%), identified as White/Caucasian (80.1%), completed post-secondary education (67%), and were employed for wages (64.2%).

**Table 1:** Demographic characteristics of women with PCOS living in Canada (*n* = 296)

Demographic characteristic	Number of women (%)
Mean ± SD age in years	29 (6)
Median (interquartile range) BMI (kg/m^2^)	33 (11)
Born overseas	31 (10.9)
Province of residence	
Alberta	48 (16.4)
Ontario	170 (58)
British Columbia	27 (9.2)
Quebec	11 (3.8)
Nova Scotia	10 (3.4)
Saskatchewan	10 (3.4)
Other	17 (5.8)
Marital status	
Single	105 (35.8)
Married or domestic partnership	183 (62.5)
Divorced, separated, or widowed	5 (1.6)
Ethnicity	
White/Caucasian	234 (80.1)
Asian/Pacific Islander	24 (8.2)
Black	7 (2.4)
Indigenous	6 (2.1)
Hispanic/Latino	2 (0.7)
Other	19 (6.5)
Education level	
High school diploma	35 (11.9)
Trade/technical/vocational training	27 (9.3)
Associate degree	36 (12.3)
Bachelor’s degree	98 (33.4)
Postgraduate degree	25 (8.5)
Professional degree	10 (3.4)
No formal qualification	62 (21.1)
Work status	
No paid work	35 (11.9)
Student	48 (16.4)
Employed for wages	188 (64.2)
Self-employed	22 (7.5)

### Diagnosis experience

The results indicate that 71.3% of respondents were diagnosed within 5 years of conducting the survey. For 34% of women, diagnosis took > 2 years from first seeing a health professional about symptoms and 41% saw 3 or more health professionals before diagnosis. Most women (65.9%) reported being dissatisfied with the information provided at diagnosis. A significant proportion reported receiving no information about lifestyle management (41.9%), and of those women who did, 33.5% were dissatisfied or very dissatisfied with the information. 38.1% of women reported being dissatisfied or very dissatisfied with information given about medical therapy, and 28.1% received no information at all. Finally, 58.8% received no emotional support or counselling after diagnosis, with 27.7% of those who did being dissatisfied or very dissatisfied with it (see Table 2).

**Table 2:** PCOS diagnosis experience among women living in Canada (*n* = 296)

Perceptions of PCOS diagnosis experience	Number of women (%)
Time since diagnosis (years)	
≤ 1.0	109 (39.5)
1.1–5.0	88 (31.8)
5.1–10.0	49 (17.8)
> 10.0	30 (10.9)
Time until diagnosis	
Within 6 months	133 (44.9)
Within 12 months	42 (14.2)
Within 2 years	20 (6.8)
More than 2 years	101 (34.1)
Number of health professionals seen before diagnosis	
1–2	175 (59)
3–4	102 (35)
≥ 5	19 (6)
Satisfaction with diagnosis experience	
Dissatisfied or very dissatisfied	111 (37.5)
Neither	65 (22)
Satisfied or very satisfied	120 (40.5)
Satisfaction with information given about PCOS	
Dissatisfied or very dissatisfied	195 (65.9)
Neither	37 (12.5)
Satisfied or very satisfied	64 (21.6)
Satisfaction with information given about lifestyle management	
Dissatisfied or very dissatisfied	99 (33.5)
Neither	33 (11.1)
Satisfied or very satisfied	40 (13.5)
This information was not mentioned	124 (41.9)
Satisfaction with information given about medical therapy	
Dissatisfied or very dissatisfied	113 (38.1)
Neither	36 (12.2)
Satisfied or very satisfied	64 (21.6)
This information was not mentioned	83 (28.1)
Satisfaction with emotional support and counselling after diagnosis	
Dissatisfied or very dissatisfied	82 (27.7)
Neither	22 (7.4)
Satisfied or very satisfied	18 (6.1)
This information was not mentioned	174 (58.8)

### Factors associated with PCOS diagnosis experiences

Spearman’s rank-order correlations determined several statistically significant, positive correlations (all *P* values ≤ 0.001) between ratings of diagnosis satisfaction (see Table 3). Overall satisfaction with the diagnosis was associated with satisfaction with overall information received about PCOS (r_s_ = 0.454). Overall satisfaction with information provided about PCOS was associated with satisfaction with information about lifestyle management (r_s_ = 0.768), medical therapy (r_s_ = 0.618), and emotional support and counselling (r_s_ = 0.650). Associations existed between satisfaction with information on lifestyle management and medical therapy (r_s_ = 0.650), and between information on medical therapy and emotional support and counselling (r_s_ = 0.635).

**Table 3:** Spearman correlation coefficients between selected variables in the study (N = 296)

Variables	Spearman correlations				
	1	2	3	4	5
1. Satisfaction with overall diagnosis	–	0.454**	0.380**	0.346**	0.351**
2. Satisfaction with info received about PCOS		–	0.768**	0.618**	0.655**
3. Satisfaction with info about lifestyle management			–	0.650**	0.583**
4. Satisfaction with info about medical therapy				–	0.635**
5. Satisfaction with emotional support and counselling					–

**p < .001

Chi-square tests determined several correlations between demographics and diagnosis experience (see Table 4). Being over 30 years of age was associated with seeing 3 or more doctors (*p* = 0.030) and waiting more than 2 years before attaining a diagnosis (*p* = 0.008). Participants who saw more than 3 doctors were more likely to wait longer than 2 years for the diagnosis (*p* < 0.001) and experience decreased satisfaction with the overall diagnosis (*p* = 0.008). Finally, residing in Ontario was associated with greater satisfaction with information provided on lifestyle management compared to all other provinces (*p* = 0.028). No significant associations were found between diagnosis experience and BMI, education, employment, and ethnicity.

**Table 4:** Chi-square results between socio-demographics and experience of diagnosis

Variables	Time to diagnosis				Number of doctors seen				Satisfaction with overall diagnosis				Satisfaction with info received on PCOS				Satisfaction with info received on lifestyle management			
	< 2 years n (%)	> 2 years n (%)	Total n (%)	X^2^; *p* value	< 3 doctors n (%)	> 3 doctors n (%)	Total n (%)	X^2^; *p* value	Satisfied n (%)	Dissatisfied n (%)	Total n (%)	X^2^; *p* value	Satisfied n (%)	Dissatisfied n (%)	Total n (%)	X^2^; *p* value	Satisfied n (%)	Dissatisfied n (%)	Total n (%)	X^2^; *p* value
Age																				
< 30 years old	108 (65.1)	58 (34.9)	166 (100)	7.016; .008*	106 (63.9)	60 (36.1)	166 (100)	4.701; .030*	71 (53)	63 (47)	134 (100)	.197; .657	37 (25.5)	108 (74.5)	145 (100)	.265; .607	21 (14.9)	120 (85.1)	141 (100)	.007; .935
> 30 years old	62 (49.6)	63 (50.4)	125 (100)		64 (51.2)	61 (48.8)	125 (100)		47 (50)	47 (50)	94 (100)		25 (22.7)	85 (77.3)	110 (100)		17 (14.5)	100 (85.5)	117 (100)	
Province																				
In Ontario	97 (57.1)	73 (42.9)	170 (100)	.703; .402	100 (58.8)	70 (41.2)	170 (100)	.015; .904	66 (50)	66 (50)	132 (100)	.468; .494	38 (26.2)	107 (73.8)	145 (100)	.397; .529	29 (19.5)	120 (80.5)	149 (100)	4.824; .028*
Outside Ontario	78 (61.9)	48 (38.1)	126 (100)		75 (59.5)	51 (40.5)	126 (100)		54 (54.5)	45 (45.5)	99 (100)		26 (22.8)	88 (77.2)	114 (100)		11 (9.6)	103 (90.4)	114 (100)	
Number of doctors seen																				
< 3 doctors n(%)	140 (80)	35 (20)	175 (100)	77.214; < .001**	–	–	–	–	79 (59.4)	54 (40.6)	133 (100)	6.971; .008*	37 (24.2)	116 (75.8)	153 (100)	.056; .813	23 (14.6)	134 (85.4)	157 (100)	.095; .758
> 3 doctors n(%)	35 (28.9)	86 (71.1)	121 (100)		–	–	–		41 (41.8)	57 (58.2)	98 (100)		27 (25.5)	79 (74.5)	106 (100)		17 (16)	89 (84)	106 (100)	

*p < 0.05

**p < 0.01. No significant associations were found between ethnicity, education level, BMI, employment, and experiences of diagnosis

Not receiving information on lifestyle management was associated with also not receiving information on medical therapy (*p* < 0.001) and emotional support and counselling (*p* < 0.001) (See Table 5). Not receiving information on lifestyle management was associated with greater dissatisfaction with overall diagnosis (*p* = 0.004) and information received about PCOS (*p* < 0.001). Missing information on medical therapy was also associated with greater dissatisfaction with the diagnosis (*p* = 0.033) and information received about PCOS (*p* = 0.001). Finally, participants who did not receive emotional support or counselling were also more likely to be dissatisfied with the overall diagnosis (*p* = 0.015) and information received about PCOS (*p* = 0.002), suggesting that these individual factors may be important to the diagnosis experience.

**Table 5:** Chi-square results between selected variables and experience of diagnosis

Variables	Satisfaction with overall diagnosis				Satisfaction with info received on PCOS				Received info on medical therapy				Received emotional support and counselling			
	Satisfied n (%)	Dissatisfied n (%)	Total n (%)	X^2^; *p* value	Satisfied n (%)	Dissatisfied n (%)	Total n (%)	X^2^; *p* value	Yes n (%)	No n (%)	Total n (%)	X^2^; *p* value	Yes n (%)	No n (%)	Total n (%)	X^2^; *p* value
Received info on LM																
Yes	82 (59.9)	55 (40.1)	137 (100)	8.430; .004**	54 (36.7	93 (63.3)	147 (100)	26.418; < .001**	152 (88.4)	20 (11.6)	172 (100)	52.972; < .001**	106 (61.6)	66 (38.4)	172 (100)	68.123; < .001**
No	38 (40.4)	56 (59.6)	94 (100)		10 (8.9)	102 (91.1)	112 (100)		62 (50)	62 (50)	124 (100)		17 (13.7)	107 (86.3)	124 (100)	
Received info on medical therapy																
Yes	94 (56.3)	73 (43.7)	167 (100)	4.547; .033*	56 (30.4)	128 (69.6)	184 (100)	11.192; .001**	–	–	–	–	116 (54.2)	98 (45.8)	214 (100)	50.911; < .001**
No	26 (40.6)	38 (59.4)	64 (100)		8 (10.7)	67 (89.3)	75 (100)		–	–	–		7 (8.5)	75 (91.5)	82 (100)	
Received emotional support and counselling																
Yes	59 (61.5)	37 (38.5)	96 (100)	5.952; .015*	36 (35.0)	67 (65.0)	103	9.640; .002**	–	–	–		–	–	–	–
No	61 (45.1	74 (54.9)	135 (100)		28 (17.9)	128 (82.1)	156 (100)		–	–	–		–	–	–	

*p < 0.05

**p < 0.01. No significant associations were found with demographic variables such as age, province, ethnicity, education level, BMI, employment

### Key concerns about PCOS

**Fig. 1:**
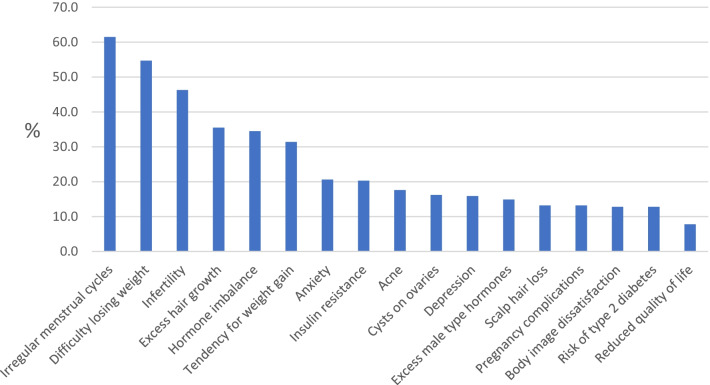
Key clinical features of PCOS most important to participants

Women were asked to select “the four key clinical features of PCOS that are most important to you.” Figure 1 displays top 17 concerns most selected by participants (%). Overall, irregular menstrual cycles (61.5%), difficulty losing weight (54.7%), infertility (46.3%), and excess hair growth (35.5%) were the most selected features. Hormonal and metabolic PCOS features were among the top concerns for participants. Features affecting mental health and well-being were substantially picked: anxiety (20.6%), depression (15.9%), body image dissatisfaction (12.8%), and reduced quality of life (7.8%). Other answer options included migraines (6.8%), ovarian cancer (6.1%), endometrial cancer (5.4%), improvement of symptoms after weight loss (4.7%), premenstrual syndrome (4.4%), increased cardiovascular risk factors (4.4%), and fatty liver (4.1%) (Fig. 1).

### Findings

The interview sample of 25 participants included mostly White/Caucasian women mainly born in Canada, with a few born in the US but residing in Canada for a long period of time (please see Table 6 for demographic characteristics). Most participants were between the ages of 25–30, resided in Ontario, and were employed full-time. Seven participants had children, and nine participants were looking to conceive at the time of the interview. All participant names presented in this article are pseudonyms.

**Table 6:** Demographic characteristics of interview participants (*n* = 25)

Demographic characteristic	Number of women (%)
Age group	
18–24	5 (20)
25–30	10 (40)
31–36	4 (16)
37–40	2 (8)
41–50	2 (8)
51–66	1 (4)
Province of residence	
Alberta	4 (16)
British Columbia	4 (16)
Ontario	13 (52)
Quebec	1 (4)
Ethnicity	
Black	1 (4)
East Asian	2 (8)
Middle Eastern	2 (8)
South Asian	2 (8)
White/Caucasian	18 (72)
Marital status	
Single	11 (44)
Common-law/live-in partner	5 (2 0)
Married	9 (36)
Education level	
Bachelor’s degree	12 (48)
Master’s degree	3 (12)
Trade/technical/vocational training	2 (8)
Parents or looking to conceive	
No children	18 (72)
Has children	7 (28)
Looking to conceive^a^	9 (36)
Pregnant	2 (8)
Work status	
No paid work	3 (12)
Student	7 (28)
Employed full-time	12 (48)
Employed part-time	1 (4)
Self-employed	2 (8)

Based on information participants were comfortable divulging, not all participants are captured

^a^Participants who were actively looking to conceive at the time of the interviews

**Fig. 2:**
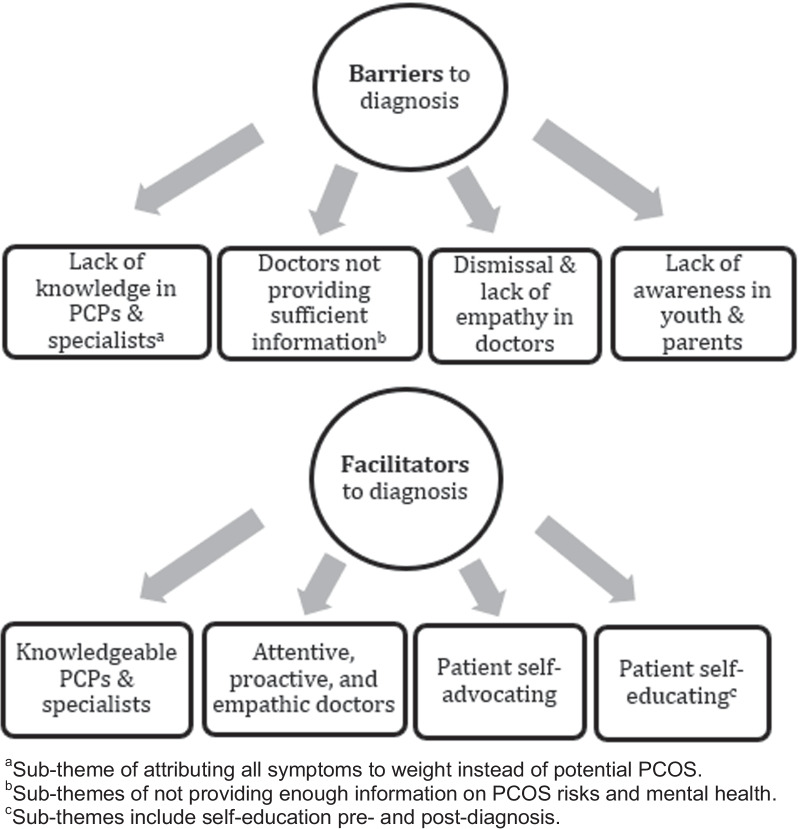
Barriers and facilitators to satisfactory diagnosis experience

Barriers were identified as factors external or internal to participants which affected the experience of the diagnosis negatively (e.g., by delaying diagnosis or worsening the experience of the diagnosis). Facilitators were identified as factors external or internal to participants which affected participant’s experience of the diagnosis positively (e.g., by quickening diagnosis or positively affecting participant experience). Figures 1 and 2 outline major themes found.

### Barriers to a satisfactory diagnosis experience

#### Lack of knowledge in primary care physicians (PCPs)

One of the biggest barriers to a prompt and accurate diagnosis of PCOS arose when participants encountered physicians who appeared to lack sufficient knowledge of PCOS. Many participants described instances where physicians failed to identify the significance of symptoms brought up, did not order tests for their patients to rule out PCOS and/or other conditions, and even missed opportunities to refer the patient to a specialist, such as a gynecologist or endocrinologist. Participants who had physicians who lacked knowledge about PCOS would experience a lot of confusion and face delayed diagnoses, waiting months to years at a time.

Josie, aged 27, recounted facing barriers with her General Practitioner (GP) in British Columbia. Josie experienced menstrual irregularities since adolescence, but her GP had not seemed concerned with any symptoms Josie would bring up. Finally, after many years of frustrating dismissals, Josie convinced her GP to refer her to a gynecologist. Josie described:

*I just had enough dealing with my women problems, whatever you want to call it, my menstrual problems, that kind of stuff. I had been trying to get my doctor to figure stuff since I was 15. 14 or 15 I knew there was something wrong because I’d never had a regular cycle since I hit puberty, started my period at 12 years old. Never had a regular cycle. I always thought it was just me, I know my mom has some sort of blood clotting disorders that she’s been diagnosed with. And I thought maybe I was susceptible to blood clotting issues, you know. My doctor never really was concerned about anything, it didn’t seem to me that way. Finally, when I was 24 I told her like “I just need you to send me to a gynecologist and to help me.” And that’s what she did. So, I got myself referred to a gynecologist at 25 actually. And then I got an IUD to help with my cycles.*

Although Josie’s GP was well-meaning, she appeared to have inadequate knowledge about PCOS to help with a diagnosis. Josie explained:

*I just struggled with my cycles and stuff, and she never seemed concerned about anything really. I guess being on birth control, some doctors don’t like women to be on that if they don’t have to be. She just kind of avoided everything possible before finally referring me to a gynecologist. I don’t know what the reasoning was behind it, I never really asked. She just seemed to avoid everything. Maybe she doesn’t know much about PCOS.*

A common sub-theme occurred within participants who were overweight – some doctors assumed that symptoms (e.g., irregular periods) were caused by excess weight and did not test for PCOS. Emma, aged 29, was diagnosed by accident after not being able to feel the strings of her IUD and going in for an ultrasound to locate the IUD. Her doctor noticed cysts on her ovaries which spurred him to diagnose her with PCOS. For years prior, Emma had experienced significant and unexplained weight gain, facial hair, and irregular periods, but when she brought up her concerns, her doctors attributed a lot of them to her weight. Emma explained:

*I gained a severe amount of weight, I gained I think.... I was 115 pounds when I was 18 and by 23, I was 200 pounds. So clearly looking back at my diet it wasn't great, but it wasn't that severe of a diet shift. So, I just assumed this is what I have, this is the body I have. And it was only because I had the ultrasound for something else that they diagnosed me. Had I not had that ultrasound, I think I would still have the cysts, I just don't think they would have diagnosed me with them, they would have kind of "oh yeah you're just doing this and this" and "oh yeah you're hysterical" or whatever. "That's just how your body is, try diet and exercise." Which is funny, because as soon as I went on metformin, I lost 30 pounds.*

PCOS occurs on a spectrum; some participants can be lean and still have PCOS. Doctors who misattributed their patient’s weight to be the cause of symptoms may not have had enough knowledge on PCOS presentation. Lucy, aged 27, was another participant who expressed frustration at her GP delaying diagnosis due to the lack of knowledge on PCOS presentation:

*Well, this is the most common reaction was “you're overweight and that's the problem.” Honestly, I feel that any time any of those symptoms were brought up, the only focus was on my weight. Rather than it being a symptom, that's the cause of all my health problems.*

To sum up, a common barrier to diagnosis for participants occurred when PCPs lacked sufficient knowledge on PCOS to identify key symptoms and test for PCOS. For overweight participants who faced this barrier, unknowledgeable PCPs posed a barrier when they overly attributed all their symptoms on weight instead of testing for PCOS.

#### Physicians not providing sufficient information

A lot of participants described needing more information at the diagnosis than they had received. They left without fully understanding the impact of PCOS, what their futures might look like, or what complications they may need to look out for. In addition, many were diagnosed but not informed about the specifics of their condition. In some cases, tests to check for insulin resistance or hyperandrogenism were not conducted, and if they were, some patients were not informed of the results of those tests.

Bianca, aged 36, was diagnosed after trying to conceive but not getting any periods after coming off birth control. Although Bianca had a great experience in finding a knowledgeable GP who quickly diagnosed her in 3 months after running some tests, she explained that missing information at the diagnosis still posed a barrier to her feeling well-equipped to deal with her condition. She reflects on how she needed more information than what she was offered:

*The most negative part of the diagnostic experience was probably the lack of answers and information. So, it was “let's do this test, let's do that test” but there wasn't a whole lot of talking it through. And with something like PCOS, I had no idea about it. I think a lot of people don't, unless they know somebody or are somebody who is dealing with it. So I think communication at the outset was lacking and I think that's probably the worst part of it because when you're dealing with the unknown when you're trying to figure this out… for me the more information the better. And I didn't feel like I was given as much as I potentially could have been.*

Many participants were not told of the full expanse of what PCOS may impact. Excerpts of the responses provided by three of the participants are as follows:

*Jamila, aged 26: “Like recently I found out that insulin resistance is one of the symptoms of PCOS. But I didn’t know it was related.”*

*Fiona, aged 31: “She didn't say anything to me about the potential side effects because I don't even know if she knew about them or if she did, I don't know. She never mentioned anything.”*

*Vanessa, aged 63: “He just said I probably couldn't have children. That was about it.”*

Participants who encountered physicians who provided little information to them would leave the doctor’s office ill-equipped to manage their condition when they were not even fully aware of its significance. Holly, aged 29, looking back on her diagnosis, felt that the lack of information provided to her led her to not take her condition as seriously and start on management. She explained:

*So, if my doctor had communicated to me that I might have trouble having kids in the future, I might have taken it more seriously when I was first diagnosed.*

Margaret, aged 33, received little information from her first GP who diagnosed her.

Margaret was able to find another GP many years later who was able to give her more information and guidance. But one thing was still missing: information about how PCOS can impact mental health. Margaret explained:

*I wish she would have maybe talked about more like the risk. I just kind of like those little satellite things that are kind of offshoots because I think that those tend to be more of the symptoms that I deal with. Like it's the higher risk for depression and anxiety and that's something that I have trouble with. Particularly the anxiety. Or like I sort of mentioned before, the tendency to binge eat because I have that problem as well. And so those were things that I didn't realize were related until I started looking into it a little bit deeper.*

In fact, Margaret was not the only participant whose doctors did not touch on mental health. Many participants had doctors who not only did not offer information on the mental health impacts of PCOS, but in general never touched on mental health during visits. Participants often discovered the link between PCOS and mental health on their own, as Margaret did during her research. Down the line, a lack of information at the diagnosis can impact how successfully women can manage their PCOS. If a patient is presented with an incomplete picture of their condition, they can go for many years underestimating the management efforts they may need to undertake.

#### Dismissal and lack of empathy in physicians

Another barrier in the diagnosis experience arose when participants encountered little empathy from doctors. Feelings of being dismissed, not listened to, and talked over were prominent and led to a perception of low empathy and lack of concern from doctors. Low levels of knowledge on PCOS may have contributed to a doctor’s dismissal of concerns, but the problem was compounded in many cases by apparent lack of empathy and unwillingness to really listen to and understand patient concerns. In many cases, lack of empathy and dismissiveness significantly affected doctor-patient interactions and trust levels.

Lizzie, aged 27, was 12 when she initially brought up her symptoms and concerns to her pediatrician, and each time her concerns were dismissed, leaving her at a loss. She explained:

*Just being as young as I was and not knowing what was going on, and them not even knowing how to explain it. Every time I would go into the doctors, and they’d be like “nothing’s wrong” I’d leave thinking “did they not tell him how bad it is? Like am I covering it up, am I imagining it worse than it really is?” So yeah, that was the biggest thing, “well what am I doing wrong? Why does nobody want to help me?”*

Lizzie was later diagnosed at age 16 when she switched doctors. Her initial GP provided little information, but mostly the lack of empathy to Lizzie’s concerns at the time affected her experience and left her feeling unsupported.

Josie, aged 27, also did not receive the level of support that she would have liked from her doctor and perceived her doctor to put little effort into understanding Josie’s experience. Josie explains her thoughts and feelings at the time:

*For me, I didn't really know what to do. I didn't really know what PCOS was. I had kind of questioned my doctor’s like willingness to help and dig deeper into my issues. For the most part, she doesn’t really dive deep into finding out why the issues are happening… With my GP, I didn’t feel very supported at all. I didn’t feel like she really tried very hard to figure anything out. Whether that was not enough time, maybe she didn’t really suspect PCOS or… you know I’m giving her the benefit of the doubt in that situation. I didn’t feel supported.*

Holly’s, aged 29, doctor was not knowledgeable about PCOS and who did not seem to take her seriously and dismissed Holly’s concerns:

*I just felt that she didn't care - not that she didn't care because I know doctors don't necessarily always have the best bedside manner. But I felt like she didn't take me seriously and that she kind of brushed off what was going on with me.*

This theme of diminished empathy and brushing off patient concerns was prevalent in many participants’ experiences and a significant barrier in the experience of receiving a diagnosis. Doctors who did not take the time to truly listen to and understand patient concerns displayed a lack of empathy which left many participants unsupported during a health challenge.

#### Age considerations: lack of awareness in youth and parents

Patients who experienced PCOS symptoms from a young age would often recall not knowing that their symptoms were abnormal or something to be concerned about at the time. The lack of awareness and concern over early symptoms led many younger participants to underrecognize the significance of certain symptoms and not bring it up at appointments, contributing to delayed diagnosis. This lack of awareness was understandable as none of the participants (or their parental figures) recalled being educated on PCOS and women’s health conditions throughout their schooling years or in mainstream media.

Margaret, aged 33, officially started to notice her symptoms and became concerned over them in her early 20s, when her periods became very irregular and she noticed unusual hair growth, which led her to reach out to health professionals. However, looking back she began experiencing odd symptoms at puberty, but recalled not being able to recognize them as concerning due to her own lack of knowledge:

*I would have been like 22 or 23-ish. I was young. I remember being young. And yeah, like I said that was kind of when I really started to notice that there was something off. And if I really sort of think back to when symptoms started, it really would have been obviously around puberty. I sort of gained a whole bunch of weight. I think I packed on like 60 pounds in a year or so. But so much of that I think got played off as just kind of being a teenager. But now kind of looking back on it, it's just like oh no, this was kind of when those symptoms really started to present themselves. I just didn't have any knowledge of it at the time.*

Margaret’s mom was coincidentally a nurse, but she also did not have enough knowledge to guide her daughter to the physician’s office for examination. Margaret recalled:

*Yeah, I don't really know why that would have been something that didn't get brought up at the time. Particularly like my mom is a nurse, you’d think she would mention it. But I mean I don't know. Maybe she was just hoping that it wouldn't be something that affected me, kind of a thing.*

Vanessa, aged 63, had heavy, painful periods ever since puberty which she started a bit early at 11. She remembered bringing up her menstrual problems to her stepmom, but her concerns went unrecognized. Vanessa recounted the interaction:

*And she kind of just brushed it off like “whatever.” To her it was just that was the word for having really bad periods. So, I kind of felt like I was making too big of a deal about it so I let it go for about ten years. And didn't really find out what it was until I was 30.*

Many participants in general had little awareness of PCOS and other women’s health conditions before becoming diagnosed. Emma, aged 29, reflected on why PCOS might not be taken seriously:

*It's just- I feel like it's either seen as no big deal because it's very common or it's just not taken very seriously because either it's a woman's issue or it's just... there are so many other issues that are more glamorous and kinder of take the forefront that people actually want to focus on. But I don't really know.*

To sum up, many participants did start having concerning symptoms early in youth, but their own lack of awareness about their symptoms and women’s health conditions in general caused a barrier to seeking medical care and achieving a diagnosis. Not being aware of PCOS and similar women’s health conditions in youth led many participants to not realize the potential significance of their symptoms and delay going in for a check-up. Similarly, the parental figures of many younger participants were not concerned about their child’s symptoms, again mostly due to a lack of knowledge. In these cases, parents were not able to encourage their daughters to visit the doctor.

### Facilitators to a satisfactory diagnosis experience

#### Knowledgeable PCPs and specialists

One of the facilitators to the diagnosis was the availability of knowledgeable doctors who were able to quickly diagnose PCOS once the patient presented their symptoms and provide enough information about PCOS to their patient. Some doctors were able to diagnose solely based on the symptoms and some ran further tests. As the first point of contact in the healthcare system, PCPs who were knowledgeable about PCOS acted as great facilitators to a speedy diagnosis, because they did not need to refer patients out and add weeks/months of waiting time.

Pam, aged 28, was one participant who had a great experience of achieving a quick diagnosis due to having a very knowledgeable GP at her local university clinic. After coming off birth control in her late 20’s she found that her period ceased to occur without it. Pam recounted:

*So, when I went to my appointment and explained that I hadn’t had a period in a year, they suggested I go for some blood tests and get all my systems checked up. Just to make sure all the levels were normal in my blood… Once I brought it up, they gave me a diagnosis within 2 weeks of that first appointment.*

Pam was able to find a team of physicians who identified the significance of her symptoms and were able to diagnose her. Pam appreciated the amount of information she was able to receive as well:

*They gave me a whole bunch of handouts to read about PCOS...I think it’s just my personality that I wanted a lot of information. So yeah, they gave me enough information but I was just presented with this new thing that I didn’t know anything about so I just wanted to learn as much as I could so I could understand what I was up against.*

In cases where PCPs had to refer patients out to see specialists (e.g., OB-GYNs, fertility doctors, endocrinologists), knowledgeable specialists were a great facilitator to diagnosis because they would be able to quickly diagnose PCOS and could provide a lot of information about PCOS at the time of diagnosis.

Josie, aged 27, had a very tough time getting her GP to take her symptoms seriously. Since the age of 14 or 15 she felt something was wrong because of her irregular cycles. Her GP *“never really was concerned about anything, it didn’t seem to me that way.*” At age 24, she felt the urgency to push her GP to send her to a gynecologist. She had started to experience cystic acne and hirsutism. At age 25, Josie’s experience with her gynecologist was very positive:

*And she actually was very helpful, she right away, she knew without asking me anything, she knew the physical signs of PCOS. She explained how PCOS works, basically explained all the symptoms. And she could tell by asking me questions and me telling her about my history, she just knew right away what it was that I had and diagnosed me with it. She was super helpful.*

To sum up, for participants who had positive experiences with their diagnosis, some of the biggest facilitators were the availability of knowledgeable PCPs and/or specialists who could provide a quick and informative diagnosis. In cases of knowledgeable PCPs, an added benefit occurred for participants who did not have experience long wait times associated with specialist referrals.

#### Attentive, proactive, and empathic doctors

Another facilitator to the diagnosis was the availability of doctors who were attentive to the concerns patients brought, proactive about providing referrals or running tests, and empathic when interacting with patients. Attentive, proactive, and empathic doctors included both PCPs and specialists and a defining feature of those doctors were their ability to start acting on a patient’s concern and provide them with resources so that they are diagnosed, even when these doctors alone did not have adequate knowledge to diagnose PCOS themselves and had to research and follow-up on symptoms.

Margaret, aged 33, had a long journey to her diagnosis—it took her 10 years to find the doctor who would be able to diagnose her. She brought up her symptoms with all the doctors but felt that they would just brush it off. She had *“given up on sort of getting anybody to really take it seriously.”* In her early 20s she came across a doctor who acknowledged that she most likely had PCOS but did not discuss it further other than prescribing birth control. Margaret moved around a lot in her 20s and felt that every time she brought up her PCOS *“it was always just kind of accepted and nobody really questioned it or really offered any kind of support.”* That is until Margaret found her latest GP.

Margaret remarked how her latest doctor *“was the first one to really highlight to me just like the importance of you know with this condition you have to be making sure that you're eating right and that you're exercising and you're at this extra risk for all of the type 2 diabetes and stuff like that.”* Margaret had seen 3–4 physicians since her early 20s and only her latest GP provided her with sufficient information and ran tests. Margaret described how her GP helped her take her condition seriously:

*The information from my most recent GP that actually did the diagnosis was probably the most helpful to me based on the fact that it kind of spurred me to really look into and kind of understand something that I kind of didn't really take seriously before.*

Josephine encountered many doctors on her journey to her diagnosis and perceived most to be unknowledgeable about PCOS. Although that aspect was frustrating for her, Josephine appreciated the empathy present in their interactions and felt supported regardless:

*The doctor in fact at the fertility clinic was very gentle and I have a very good impression with him. So, most of the time, health professionals I think are very good. But yes, sometimes they don't know about everything. But yeah, during my diagnostic, yes the doctor was good. And the people that I've seen in the hospital, they were very gentle, and I had a very good impression.*

To sum up, participants appreciated physicians who were able to display empathy and be attentive and proactive when addressing concerns. Even when physicians did not know much about PCOS, participants still felt supported when encountering their attentiveness, empathy, and proactiveness.

#### Self-advocating

In cases where participants felt that their concerns were not taken seriously and a diagnosis was evading them, a great facilitator to the diagnosis was their own ability to self-advocate—in a sense to push to be taken seriously and to get the doctor to take their symptoms seriously and/or refer them out to a specialist. Participants had to self-advocate mostly with PCPs, but some participants had to self-advocate with specialists too who were unsure of what to do. Older participants often portrayed a greater motivation to self-advocate, usually due to increasing concerns with health and aging or when pursuing conception.

Mary, aged 27, had a unique case of PCOS where her symptoms started after the birth of her first two children. Mary found that her doctors were hesitant to investigate her concerns, possibly because Mary was still breastfeeding. But Mary persisted and eventually persuaded them for a referral:

*Oh yeah, I was really really really aggressive about it. They were not - based on the blood work they were going to send me away, but I had to be. And honestly it was just because I really wanted another baby. So, I was kind of having to really advocate for myself because I knew… because they just looked at me like “okay well you're breastfeeding, so that's the reason for your issues.” Which I found in general there's a lack of knowledge for breastfeeding women and when it comes to PCOS there's some overlap there because they definitely just blamed it on “oh that's what's messing up your hormones.” Even though the hormones of breastfeeding and your period managing hormones or whatever, are not the same. So, they had a lot of confusion, I just had to really push them to kind of get that referral. The nurse that I’d seen was originally very hesitant towards referring me. Which is so crazy because if I wasn't trying to have a baby, I wouldn't know to this day that I have PCOS.*

Mary was very motivated to push for herself and advocate for her concerns because she wanted to conceive again. Her efforts to self-advocate paid off and led to a diagnosis of PCOS which allowed her to take charge of her health and aid her in her quest to conceive. At the time of the interview, Mary had become pregnant after being prescribed clomiphene. Mary explained how attaining a diagnosis finally allowed her to feel less lost and out of control and helped her get pregnant again:

*The most positive part about it was that I was able to figure out what was going on with my body and that I was able to be properly treated to get pregnant because that's really what was motivating me. I started to feel really kind of lost about not having a period and not really having it in my control. So even though I wasn't really given a lot of resources, I feel like once I knew what was going on I was kind of able to advocate for myself enough to get those resources and yeah, just getting pregnant, honestly.*

Lucy, aged 49, explains that her doctors seemed to not take her concerns seriously since she had given birth twice. One of Lucy’s sisters went through a remarkably similar experience getting diagnosed with PCOS and having to self-advocate. Lucy described:

*She had the same issue I did, in that it was very difficult. She had to fight for a diagnosis. Amazingly enough, like she's in her 30s now, it's only recently that she was diagnosed with PCOS even though she's known that she's had it for years. It just took them forever to admit it. And that was the same with me. They wouldn't even look at me initially and consider that I had PCOS because I already had two children.*

Like Lucy, many participants shared the stories they heard about other women having to self-advocate and push to get the resources they needed for the diagnosis. Self-advocating, in cases where knowledgeable and proactive doctors were unavailable, proved to be a significant facilitator in achieving a diagnosis. Attaining a diagnosis aided participants in feeling empowered and able to take charge of their health in many ways, such as by getting them started on treatments to address their symptoms, undertake the necessary lifestyle changes, and allowing them to feel less lost and out of control about what was happening to their bodies.

#### Self-education

Self-education pre- and post-diagnosis was a significant facilitator for many participants who lacked sufficient information from medical providers. Many participants benefited from a period of self-education when encountering unknowledgeable doctors who could not diagnose their symptoms. Usually frustrated with the lack of helpful information from doctors, participants started to learn more about the potential causes to their symptoms on their own. Whether it was through reading up about their symptoms online, talking to friends, or joining online mailing lists/groups, participants were able to self-educate and attain a diagnosis after presenting new knowledge to their doctors. In other cases, participants who were able to get diagnosed but did not receive enough information from their doctors benefited from self-education post-diagnosis.

Melissa had struggled with her weight all her life, and after having her first child in her 20s she struggled to get pregnant again and her periods became increasingly irregular. She experienced many miscarriages and noticed facial hair and skin tags. Melissa’s GP at the time attributed all her symptoms to her weight, instead of testing for PCOS. Melissa described:

*My GP that I had, any time I brought up any concerns about any concerns I had about my body, she would say “lose weight and you’ll be fine, you’re just overweight.” Eventually, I just had to fire her, I didn’t really feel safe with her.*

Melissa’s GP was unable to provide her with relevant information, so Melissa reached out to a friend with PCOS and learned more about her symptoms:

*I spoke to a girlfriend who had been diagnosed with PCOS and she suggested that I talk to my reproductive endocrinologist (RE) about the possibility that I have PCOS because of all the symptoms I had. So, when I went to see the RE, I mentioned to her that I was concerned that I may have PCOS because of my symptoms, my lack of ovulation which is kind of like the lack of periods. So, she sent me for assessing blood tests to check my insulin resistance, and she said based on my results and my symptoms, she was diagnosing with PCOS.*

Due to Melissa’s efforts to self-educate with the help of a friend, she learned more about what may be causing her symptoms and was able to achieve a diagnosis with an RE, despite having an unknowledgeable GP.

Bianca’s, aged 36, GP was knowledgeable enough to diagnose her with PCOS quickly. The only thing lacking from her GP was sufficient information about PCOS, but Bianca was able to delve into what it meant to have PCOS with self-education.

*I mostly did a lot of online research. First, I try to see what kind of medical articles I could get my hands on to see what's going on. Especially when I was trying to conceive and what works better for women with PCOS in terms of ovulating and things like that. But I also at the beginning spent a lot of time on the Soul Cysters web site and I find that really really good as a source of information.... It was a good collective source of information.*

Sources of information for participants varied, with many participants recognizing that not all information online was reliable, but still valuable to sort through because that was often the only source of information for participants to access. Self-education pre-diagnosis aided participants who were faced with unknowledgeable doctors and facilitated their journey to their diagnosis. Self-education post-diagnosis helped participants piece together and understand their condition better when they did not receive sufficient, or any, information from physicians.

## Discussion

Most interview participants expressed frustration at how few medical professionals they encountered had substantial knowledge and information about PCOS to provide, which ended up being a significant barrier in their diagnosis journeys. Survey findings included a majority (65.9%) reporting being dissatisfied with the information provided at diagnosis. A significant proportion of participants received no information about lifestyle management (41.9%), and of those who did, 33.5% were dissatisfied or very dissatisfied with the information. About a third (38.1%) of participants reported being dissatisfied or very dissatisfied with information given about medical therapy, and 28.1% received no information about medical therapy at all. These survey findings closely resemble the satisfaction scores from other surveys done on PCOS populations in Australia and internationally [28, 29].

During the interviews, numerous participants expressed leaving the doctor’s office none-the-wiser about the impacts of PCOS. Participants reported not learning about the associations between PCOS and insulin resistance, weight gain, and even infertility. These sub-themes may help explain why 65.9% of survey respondents were dissatisfied or very dissatisfied with the information provided to them at the time of diagnosis. Participants tended to learn about those impacts on their own, during self-education post-diagnosis. Similar findings were reported on the gaps in information patients receive about PCOS in the doctor’s office, with many recommendations to include pamphlets or other summative informational sources about PCOS so that patients can have access to credible medical information at the time of diagnosis [28–31, 39–43].

A sub-theme was identified under the larger barrier of not receiving sufficient information: participants reported physicians not screening for or acknowledging mental health. Top key features most important to participants included several mental health-related symptoms: anxiety (20.6%), depression (15.9%), and body image dissatisfaction (12.8%). A majority (58.8%) of survey participants did not receive emotional support and counselling at the time of diagnosis, and of those who did, 27.7% were dissatisfied or very dissatisfied. Almost none of the 25 interview participants recounted their medical professionals asking about their mental health or providing information about PCOS impacts on mental health. Several participants expressed needing information from their provider on the potential mental health impacts of PCOS, such as the increased risk for eating disorders [12, 14], and anxiety and depression [15–17]. For participants with eating disorders, generic lifestyle advice to eat less and exercise more to lose weight can exacerbate disordered eating. Lifestyle therapies for PCOS may benefit from shifting focus away from body weight; use of weight-neutral, non-restrictive nutrition programming has been found effective at promoting lifestyle changes while also reducing disordered eating patterns [44, 45]. Several guidelines have been published in the last 5–10 years detailing the need for mental health screening at the time of diagnosis [13, 15, 17]. Although previous studies and guidelines had been published to highlight the importance of mental health screening with PCOS, recent data from this study and others [28–30] suggests that so far that clinical shift does not appear to have occurred yet.

Self-advocation and self-education were particularly strong, and unexpected, themes to emerge from the data. Many participants described needing to advocate for themselves to convince their physicians that their symptoms were (a) real and (b) serious enough to warrant testing and/or referral. Often, participants self-advocated and self-educated concurrently; self-education sometimes informed and supported self-advocation efforts at the doctor’s office. The self-advocation and self-education themes emerged from other study population in the literature [30, 31, 46, 47]. In this study population, it appeared that few participants had easy-going diagnosis experiences or the opportunity to be taken care of by knowledgeable doctors who were well-versed on PCOS and could provide ample support to patients at the time of the diagnosis. When such support was missing from doctors, participants’ own efforts to self-educate (pre- and post-diagnosis) and self-advocate facilitated their efforts to attain a diagnosis that explained their symptoms.

A concerning theme within participant experiences included the large number of women experiencing their concerns and symptoms being dismissed chronically by their doctors, and feeling unheard, brushed off, and not taken seriously, leading to delayed diagnoses. This theme occurred across age groups, from women being diagnosed as adolescents, young adults, and even in adult women who started to experience symptoms after childbirth. Many participants went years having their concerns dismissed, and diagnosis delayed, until they switched doctors or began to self-advocate. For overweight participants, their concerns and symptoms were often brushed off as being due to their weight instead of potential PCOS, a finding that was also reported by a recent qualitative study [48]. Similar findings of dismissal and lack of empathy from physicians were reported by several other studies [30, 31, 43, 47]. Some participants attributed their physician’s dismissals on a lack of knowledge about PCOS; other participants felt that chronic and widespread dismissal of PCOS symptoms fell under systemic under-prioritization and unawareness of women’s health. In cases with dismissive doctors, participants often benefitted from seeking counsel from another doctor, such as by changing family doctors or seeking referrals. Self-advocation was a particularly strong facilitator for participants whose concerns were brushed off. Participants would “push” to be taken seriously and continue advocating for themselves until something was done to address their concerns.

One of the biggest facilitators in patient journeys was the availability of knowledgeable PCPs who were able to diagnose patients quickly without the need for referral, allowing participants to be informed of their health status quickly and get them started on their management journeys. Chi-squares revealed that participants who saw more than 3 doctors before attaining diagnosis were more likely to wait more than 2 years for the diagnosis and be less satisfied with the overall diagnosis. Previous studies point to a widespread gap in awareness of PCOS in health professionals. Surveys of OB-GYN residents and physicians found deficiencies in knowledge of PCOS diagnostic criteria where only 55% of trainees correctly identified the 3 main Rotterdam criteria despite 85.4% reporting using the criteria to diagnose PCOS [26]. Another study found that most physicians surveyed reported not knowing which PCOS diagnostic criteria they used [27], pointing to a need for greater physician training so that the care they deliver for PCOS aligns with national and international guidelines [26, 27]. Participants may benefit the most from knowledgeable PCPs in Canada, as it can cut down wait times associated with referrals and the number of doctors they need to see before starting on treatment. Special targeting for PCP education may be needed in Canada and countries with similarly structured healthcare systems.

In terms of age considerations, a few themes occurred for participants who were diagnosed in adolescence. Reflecting, participants found a general lack of awareness of PCOS and other women’s health conditions within themselves in adolescence and in parental figures. Due to lack of awareness, many adolescents with symptoms would fail to recognize the potential implications and not bring up their symptoms at doctor’s offices. Youth were often not able to self-advocate compared to older participants, who emphasized the need for self-advocation at the doctor’s office. Several studies found little awareness of PCOS in students internationally [49, 50] and a need for awareness programmes [51, 52]; however, more research is needed to gauge awareness levels in adolescents in Canada.

More commonly, adolescents would bring up symptoms but would encounter physicians who dismissed symptoms and were unable to diagnose. Although participants may have attributed the lack of action their physicians took to diagnose them in adolescence to lack of knowledge, physicians may be purposely delaying diagnosis avoid overdiagnosis in adolescents as PCOS symptoms often overlap with normal pubertal development, instead opting to consider them as “increased risk” as recent guidelines suggest [53]. However, with many youth reporting dismissals and a lack of information from doctors, physicians in these cases may need to ensure that they adequately communicate why they may be delaying diagnosis if that is the case. Education for youth to be able to identify potential symptoms of PCOS may be necessary, along with greater education for physicians on how to navigate providing care for adolescent populations [43].

The findings laid out in barriers and facilitators to the diagnosis experience ultimately attempt to add to a rich body of evidence to provide useable knowledge to clinicians and stakeholders and optimize clinical responses for PCOS healthcare. Many patients with PCOS do not receive adequate medical guidance and information, contributing to delayed and unsatisfactory diagnoses, as well as possible loss of trust [54]. Findings from this paper resemble previous findings about women’s experience of PCOS diagnosis from as far back as 21 years ago (at the turn of the century) [55] suggesting that although research has been conducted with this population, and recommendations have been laid out, clinical response has not yet been optimized for this population. Greater awareness and research into this population’s experience with healthcare delivery is necessary to provide women with equitable healthcare.

### Limitations and strengths

Limitations of this study include self-reported diagnosis of PCOS, potential for recall bias, and selection bias. Recall bias is possible for participants who received their diagnosis many years prior to completing the survey. Selection bias is possible due to all participants being recruited from websites and online groups, eliminating the potential to reach women with PCOS who are not as active online. The questionnaire was also only available in English and posted on English web sites, and thus, may have excluded non-English speaking populations, such as immigrant populations. Most survey and interview participants identified as Caucasian; few participants from diverse ethnic backgrounds were captured. The sample may not be representative of the general population of women with PCOS, and limited conclusions can be drawn regarding other world regions. The data gathered is largely consistent with previous research internationally and in North America [28, 29]. Member checking, where participants are given an opportunity to review findings and provide feedback, was not performed since participant contact information was not recorded and permission was not sought to save information and contact the interviewees when data analysis was complete.

With regards to the interviewer’s positionality, MI shared with participants her prior knowledge about PCOS due to having friends with PCOS, her personal interest in the topic, but that she herself did not have a PCOS diagnosis. Since MI herself did not have PCOS, participants may have felt more reserved than if they had been interviewed by someone with PCOS; however, disclosing that MI was personally connected to friends who do, may have helped participants feel more understood. As MI was a student and novice researcher, who was not involved in the healthcare profession, participants may have felt more at ease being open about their experiences navigating the healthcare system without the presence of a potential power dynamic.

Strengths of this study include involvement of qualitative research to help clarify and gather context on survey results. Previous survey-based studies were solely quantitative and may have failed to capture the richness of data from qualitative research [28, 29]. This study addressed the need for qualitative research to compliment the results of survey data [29]. Another strength of this study was the involvement of peri- and post-menopausal women whose experiences and beliefs may not be transferable from the experiences of women of reproductive age. Previous qualitative studies largely involved women under 50 years of age [30, 31, 41, 47].

## Conclusion

Poor experiences with attaining diagnosis revealed numerous barriers faced by women with PCOS. There are clear opportunities for improving patient experiences: improving PCOS awareness in medical professionals (particularly at the PCP level) and in the general population, promoting the use of diagnosis guidelines and recommendations in clinical settings, and the provision of credible medical information at the time of diagnosis. Women appeared to benefit most from physicians who were knowledgeable and informative; however, attentive physicians who investigated and addressed patient concerns even with little knowledge on PCOS aided women’s journey to their diagnosis. Although self-advocation aided participants greatly in their journeys, it might be time for patients with PCOS to rest easy knowing that their physicians will be healthcare advocates on their behalf.

### Policy recommendations and future research

Participant experiences revealed that some things are amiss with PCOS healthcare delivery and clinical shifts are necessary to provide appropriate care for this complex condition. PCOS awareness, and perhaps awareness of overall women’s health issues, may need greater integration in medical curriculums and resident training, in primary care especially. Advocation to relevant peak bodies, stakeholders, and professional medical societies is needed to raise awareness of the potential need for educational reform on PCOS for health professionals in Canada. Several international evidence-based guidelines and reviews [2, 20, 22, 33, 53] have been established for the diagnosis and management of PCOS which can be consulted by clinicians in practice. The guidelines inform of the necessary information to provide and of the importance of mental health screening, among other screening and testing to be done, accounting for the needs of different phenotypes and age groups of women. Evidence-based translation and education resources for physicians and women with PCOS, along with the first, evidence-based app for women with PCOS “AskPCOS,” can be found at https://www.monash.edu/medicine/sphpm/mchri/pcos/resources. These translation resources were developed in conjunction with health professionals and women with PCOS.

Future research is needed to explore the experiences of women from various ethnic backgrounds accessing care for PCOS in Canada, a country with many immigrant women who may experience greater barriers to care not captured in this study. Studies are also needed to explore rural populations who were missed by this study, who live with limited access to doctors and who may also face greater barriers to care in Canada. Studies on physician populations are also needed to gauge PCOS awareness and inform education efforts. Finally, further studies are needed with women outside of reproductive age, such as peri- and post-menopausal women with PCOS whose experiences accessing are still not fully understood.

## Supplementary Information

**Additional file 1.** Interview guide.

**Additional file 2**. CHERRIES checklist.

**Additional file 3.** COREQ checklist.

## Data Availability

The interview guide containing questions about the diagnosis is made publicly available, other data are available from the corresponding author on reasonable request.

## Abbreviations

COREQ: Consolidated criteria for reporting qualitative research
GP: General practitioner
OB-GYN: Obstetrician-gynecologist
PCOS: Polycystic ovary syndrome
PCP: Primary care physician

## References

[1] Bozdag G, Mumusoglu S, Zengin D, Karabulut E, Yildiz BO (2016). The prevalence and phenotypic features of polycystic ovary syndrome: a systematic review and meta-analysis. Hum Reprod.

[2] Teede H, Deeks A, Moran L (2010). Polycystic ovary syndrome: a complex condition with psychological, reproductive and metabolic manifestations that impacts on health across the lifespan. BMC Med.

[3] Balen AH, Morley LC, Misso M, Franks S, Legro RS, Wijeyaratne CN (2016). The management of anovulatory infertility in women with polycystic ovary syndrome: an analysis of the evidence to support the development of global WHO guidance. Hum Reprod Update.

[4] Lim SS, Davies MJ, Norman RJ, Moran LJ (2012). Overweight, obesity and central obesity in women with polycystic ovary syndrome: a systematic review and meta-analysis. Hum Reprod Update.

[5] Ollila MME, West S, Keinänen-Kiukaanniemi S, Jokelainen J, Auvinen J, Puukka K (2017). Overweight and obese but not normal weight women with PCOS are at increased risk of Type 2 diabetes mellitus: a prospective, population-based cohort study. Hum Reprod.

[6] Baranova A, Tran TP, Birerdinc A, Younossi ZM (2011). Systematic review: Association of polycystic ovary syndrome with metabolic syndrome and non-alcoholic fatty liver disease. Aliment Pharmacol Ther.

[7] Lim SS, Kakoly NS, Tan JWJ, Fitzgerald G, Bahri Khomami M, Joham AE (2019). Metabolic syndrome in polycystic ovary syndrome: a systematic review, meta-analysis and meta-regression. Obes Rev.

[8] Schmidt J, Landin-Wilhelmsen K, Brännström M, Dahlgren E (2011). Cardiovascular disease and risk factors in PCOS women of postmenopausal age: a 21-year controlled follow-up study. J Clin Endocrinol Metab.

[9] Wild RA, Carmina E, Diamanti-Kandarakis E, Dokras A, Escobar-Morreale HF, Futterweit W (2010). Assessment of cardiovascular risk and prevention of cardiovascular disease in women with the polycystic ovary syndrome: a consensus statement by the androgen excess and polycystic ovary syndrome (AE-PCOS) society. J Clin Endocrinol Metab Endocr Soc.

[10] Bazarganipour F, Ziaei S, Montazeri A, Foroozanfard F, Kazemnejad A, Faghihzadeh S (2013). Body image satisfaction and self-esteem status among the patients with polycystic ovary syndrome. Int J Reprod BioMed.

[11] Himelein MJ, Thatcher SS (2006). Depression and body image among women with polycystic ovary syndrome. J Health Psychol.

[12] Tay CT, Teede HJ, Hill B, Loxton D, Joham AE (2019). Increased prevalence of eating disorders, low self-esteem, and psychological distress in women with polycystic ovary syndrome: a community-based cohort study. Fertil Steril.

[13] Dokras A, Stener-Victorin E, Yildiz BO, Li R, Ottey S, Shah D (2018). Androgen Excess-Polycystic Ovary Syndrome Society: position statement on depression, anxiety, quality of life, and eating disorders in polycystic ovary syndrome. Fertil Steril.

[14] Månsson M, Holte J, Landin-Wilhelmsen K, Dahlgren E, Johansson A, Landén M (2008). Women with polycystic ovary syndrome are often depressed or anxious: a case control study. Psychoneuroendocrinology.

[15] Cooney LG, Lee I, Sammel MD, Dokras A (2017). High prevalence of moderate and severe depressive and anxiety symptoms in polycystic ovary syndrome: a systematic review and meta-analysis. Hum Reprod.

[16] Damone AL, Joham AE, Loxton D, Earnest A, Teede HJ, Moran LJ (2019). Depression, anxiety and perceived stress in women with and without PCOS: a community-based study. Psychol Med.

[17] Dokras A, Clifton S, Futterweit W, Wild R (2012). Increased prevalence of anxiety symptoms in women with polycystic ovary syndrome: systematic review and meta-analysis. Fertil Steril.

[18] The Rotterdam ESHRE/ASRM-sponsored PCOS consensus workshop group (2004). Revised 2003 consensus on diagnostic criteria and long-term health risks related to polycystic ovary syndrome (PCOS). Hum Reprod.

[19] Goodman NF, Cobin RH, Futterweit W, Glueck JS, Legro RS, Carmina E (2015). American association of clinical endocrinologists, American college of endocrinology, and androgen excess and pcos society disease state clinical review: guide to the best practices in the evaluation and treatment of polycystic ovary syndrome—part 1. Endocr Pract.

[20] Legro RS, Arslanian SA, Ehrmann DA, Hoeger KM, Murad MH, Pasquali R (2013). Diagnosis and treatment of polycystic ovary syndrome: an endocrine society clinical practice guideline. J Clin Endocrinol Metab.

[21] Actkins KV, Singh K, Hucks D, Velez Edwards DR, Aldrich M, Cha J (2021). Characterizing the clinical and genetic spectrum of polycystic ovary syndrome in electronic health records. J Clin Endocrinol Metab.

[22] Hoeger KM, Dokras A, Piltonen T (2021). Update on PCOS: consequences, challenges, and guiding treatment. J Clin Endocrinol Metab.

[23] Hardy TSE, Norman RJ (2013). Diagnosis of adolescent polycystic ovary syndrome. Steroids.

[24] Witchel SF, Oberfield S, Rosenfield RL, Codner E, Bonny A, Ibáñez L (2015). The diagnosis of polycystic ovary syndrome during adolescence. Horm Res Paediatr.

[25] Bonny AE, Appelbaum H, Connor EL, Cromer B, DiVasta A, Gomez-Lobo V (2012). Clinical variability in approaches to polycystic ovary syndrome. J Pediatr Adolesc Gynecol.

[26] Chemerinski A, Cooney L, Shah D, Butts S, Gibson-Helm M, Dokras A (2020). Knowledge of PCOS in physicians-in-training: identifying gaps and educational opportunities. Gynecol Endocrinol.

[27] Dokras A, Saini S, Gibson-Helm M, Schulkin J, Cooney L, Teede H (2017). Gaps in knowledge among physicians regarding diagnostic criteria and management of polycystic ovary syndrome. Fertil Steril.

[28] Gibson-Helm M, Teede H, Dunaif A, Dokras A (2017). Delayed diagnosis and a lack of information associated with dissatisfaction in women with polycystic ovary syndrome. J Clin Endocrinol Metab.

[29] Gibson-Helm ME, Lucas IM, Boyle JA, Teede HJ (2014). Women’s experiences of polycystic ovary syndrome diagnosis. Fam Pract.

[30] Soucie K, Samardzic T, Schramer K, Ly C, Katzman R (2021). The diagnostic experiences of women with polycystic ovary syndrome (PCOS) in Ontario, Canada. Qual Health Res.

[31] Tomlinson J, Pinkney J, Adams L, Stenhouse E, Bendall A, Corrigan O (2017). The diagnosis and lived experience of polycystic ovary syndrome: a qualitative study. J Adv Nurs.

[32] Deeks AA, Gibson-Helm ME, Paul E, Teede HJ (2011). Is having polycystic ovary syndrome a predictor of poor psychological function including anxiety and depression?. Hum Reprod.

[33] Teede HJ, Misso ML, Boyle JA, Garad RM, McAllister V, Downes L (2018). Translation and implementation of the Australian-led PCOS guideline: clinical summary and translation resources from the International Evidence-based Guideline for the Assessment and Management of Polycystic Ovary Syndrome. Med J Aust.

[34] Thorne S, Kirkham SR, O’Flynn-Magee K (2004). The analytic challenge in interpretive description. Int J Qual Methods.

[35] Statistics Canada. Analysis : population by age and sex. Annual Demographic Estimates: Canada, Provinces and Territories, 2018. 2018. https://www150.statcan.gc.ca/n1/pub/91-215-x/2020001/sec2-eng.htm.

[36] Braun V, Clarke V (2006). Using thematic analysis in psychology. Qual Res Psychol.

[37] Eysenbach G (2004). Improving the quality of web surveys: the checklist for reporting results of internet E-surveys (CHERRIES). J Med Internet Res.

[38] Tong A, Sainsbury P, Craig J (2007). Consolidated criteria for reporting qualitative research (COREQ): a 32-item checklist for interviews and focus groups. Int J Qual Health Care.

[39] Ching HL, Burke V, Stuckey BGA (2007). Quality of life and psychological morbidity in women with polycystic ovary syndrome: body mass index, age and the provision of patient information are significant modifiers. Clin Endocrinol.

[40] Avery JC, Braunack-Mayer AJ (2007). The information needs of women diagnosed with Polycystic Ovarian Syndrome: implications for treatment and health outcomes. BMC Women’s Health.

[41] Ee C, Smith C, Moran L, MacMillan F, Costello M, Baylock B (2020). “The whole package deal”: experiences of overweight/obese women living with polycystic ovary syndrome. BMC Women’s Health.

[42] Tomlinson J, Letherby G, Pinkney J, Millward A, Stenhouse E (2013). Raising awareness of polycystic ovary syndrome. Nurs Stand.

[43] Jones GL, Hall JM, Lashen HL, Balen AH, Ledger WL (2011). Health-related quality of life among adolescents with polycystic ovary syndrome. J Obstet Gynecol Neonatal Nurs.

[44] Clifford D, Ozier A, Bundros J, Moore J, Kreiser A, Morris MN (2015). Impact of non-diet approaches on attitudes, behaviors, and health outcomes: a systematic review. J Nutr Educ Behav.

[45] Pirotta S, Joham AJ, Moran LJ, Skouteris H, Lim SS (2021). Implementation of evidence-based PCOS lifestyle management guidelines: perceived barriers and facilitators by consumers using the theoretical domains framework and COM-B model. Patient Educ Couns.

[46] Satveit S (2018). Addressing the unique healthcare needs of women: opportunity for change exists at the intersection of precision health and learning health systems. Learn Health Syst.

[47] Williams S, Sheffield D, Knibb RC (2015). ‘Everything’s from the inside out with PCOS’: exploring women’s experiences of living with polycystic ovary syndrome and co-morbidities through Skype™ interviews. Health Psychol Open.

[48] Copp T, Muscat DM, Hersch J, McCaffery KJ, Doust J, Dokras A (2021). The challenges with managing polycystic ovary syndrome: a qualitative study of women’s and clinicians’ experiences. Patient Educ Couns.

[49] Haq N, Khan Z, Riaz S, Nasim A, Tahir M (2017). Prevalence and knowledge of polycystic ovary syndrome (PCOS) among female science students of different public Universities of Quetta, Pakistan. Imp J Interdiscip Res.

[50] Pramodh S (2020). Exploration of lifestyle choices, reproductive health knowledge, and polycystic ovary syndrome (Pcos) awareness among female Emirati University students. Int J Women’s Health.

[51] Lotfy Mohamed El Sayed S, Lotfy Mohamed El Sayed M, Chinedu Michael G (2020). Screening for polycystic ovarian syndrome and effect of health education on its awareness among adolescents: a pre-post study. Int J Nurs Educ.

[52] Rajkumari P, Sahoo J, Sujata P, Sahoo G, Hansa J (2016). Awareness about PCOS and the likelihood of its symptoms in adolescent girls in a semi-urban set-up: a cross sectional study. J Med Sci Clin Res.

[53] Teede HJ, Misso ML, Costello MF, Dokras A, Laven J, Moran L (2018). Recommendations from the international evidence-based guideline for the assessment and management of polycystic ovary syndrome. Hum Reprod.

[54] Lin AW, Bergomi EJ, Dollahite JS, Sobal J, Hoeger KM, Lujan ME (2018). Trust in physicians and medical experience beliefs differ between women with and without polycystic ovary syndrome. J Endocr Soc.

[55] Hacihanefioglu B (2000). Polycystic ovary syndrome nomenclature: chaos?. Fertil Steril.

