# Supplementary material for: “I felt like she didn’t take me seriously”: a multi-methods study examining patient satisfaction and experiences with polycystic ovary syndrome (PCOS) in Canada
Source: BMC Womens Health. 2022 Feb 23;22:47. doi: 10.1186/s12905-022-01630-3 (PMC8864824; doi:10.1186/s12905-022-01630-3)
Supplement: Supplementary file 1 — Additional file 1. Interview guide. [file 12905_2022_1630_MOESM1_ESM.pdf]

Interview Guide

Experiences with diagnosis:

1. Can you tell me a little bit about yourself?
2. When did you first learn about PCOS?
3. Could you tell me about how you came to be diagnosed with PCOS? (from when you first noticed something wrong)
4. Could you tell me about the treatment(s) your physician recommended for you?
5. What have been the positive aspects of your diagnostic experience?
6. What have been the negative aspects of your diagnostic experience?
7. Could you tell me about your impressions of the healthcare system at the time you were diagnosed?
8. How did the diagnostic process affect your quality of life?
