# Supplementary material for: “I felt like she didn’t take me seriously”: a multi-methods study examining patient satisfaction and experiences with polycystic ovary syndrome (PCOS) in Canada
Source: BMC Womens Health. 2022 Feb 23;22:47. doi: 10.1186/s12905-022-01630-3 (PMC8864824; doi:10.1186/s12905-022-01630-3)
Supplement: Supplementary file 2 — Additional file 2. CHERRIES checklist. [file 12905_2022_1630_MOESM2_ESM.pdf]

**Checklist for Reporting Results of Internet E-Surveys (CHERRIES)***

| *Item Category/Checklist Item* | *Explanation* | *Page Number* |
|---|---|---|
| **Design** | | |
| Describe survey design | The target population were women over the age of 18, with a self-declared PCOS diagnosis made by a medical professional in Canada, and who have resided in Canada since their diagnosis. The sample was a convenience sample drawn from online PCOS groups on Facebook, Reddit, and various PCOS online forums. | Methods, pg. 4-5 |
| **IRB (Institutional Review Board) approval and informed consent process** | | |
| IRB approval | Ethics approval for this study was received from the University of Ottawa Research Ethics Boards (REB) in April 2018. | Methods, pg. 9 |
| Informed consent | Informed consent for the survey was obtained from all those agreeing to complete the survey. The participants were informed of the purpose of the study, who the investigators were, that their responses will remain confidential and accessible only to the researchers, the length of time of the survey, and that by clicking “I agree” and starting the survey, they were declaring consent to participate but could withdraw their data at any time. Participants were informed of all this via the survey welcome page which also held a link to the full implied consent form. | Methods, pg. 9 |
| Data protection | The survey data was hosted solely on the first author’s SurveyMonkey account on Canadian servers and was password-protected. No personal information was linked to survey results in any way (e.g., contact information to enter the draw). The fully de-identified dataset is kept on password-protected computers. | Methods, pg. 9 |
| **Development and pre-testing** | | |
| Development and testing | The survey was drawn from a previous study by Gibson-Helm et al. (2017) which was developed with input from a multidisciplinary expert advisory group and piloted with women with PCOS. Permission to replicate the survey was granted by the authors and publisher in June 2018. The survey was modified by the first author to include eligibility questions about participants being diagnosed and residing in Canada. The first author tested the survey for functionality before fielding. No pilot testing was performed. | Methods, pg. 4 |
| **Recruitment process and description of the sample having access to the questionnaire** | | |
| Open survey versus closed survey | This was an open survey hosted on SurveyMonkey. | Methods, pg. 6 |
| Contact mode | Contact mode was online via advertisement posts made in PCOS groups on Facebook and Reddit and online PCOS forums. | Methods, pg. 5-6 |

| | | |
|---|---|---|
| Advertising the survey | The survey was advertised entirely online by the first author posting a short paragraph about themselves and the purpose of the study along with a recruitment poster and a link to the survey on SurveyMonkey. The same post was posted by the first author in PCOS groups on Facebook and Reddit, along with several PCOS online forums found by searching Google. The PCOS Awareness Association also helped with recruitment by doing a one-time re-post on their Facebook page with the study's recruitment poster and the survey link. | Methods, pg. 5-6 |
| **Survey administration** | | |
| Web/E-mail | SurveyMonkey survey (web-based) | Methods, pg. 4 |
| Context | SurveyMonkey is a website for constructing, storing, and analysing online surveys. The administrator can design the length, the kind of information provided and the type of questions & answers. The website appearance is neutral and not influential on the sample. | Methods, pg. 4 |
| Mandatory/voluntary | A voluntary survey. | Methods, pg. 6 |
| Incentives | Participants were informed during advertising and on the survey welcome page that if interested, they could enter a draw to win 1 of 3 CAD$25 Amazon e-gift cards which would be sent out by email. | Methods, pg. 6 |
| Time/Date | They survey was advertised, and data was collected across April-December 2018. Advertisement posts were made in the online groups in April, October, and December 2018. | Methods, pg. 6 |
| Randomization of items or questionnaires | No randomization of items was used. | N/A |
| Adaptive questioning | Adaptive questioning was used via SurveyMonkey's logic tool. Relevant survey items were displayed based on previous responses (e.g., only those who had seen multiple physicians were asked how many they saw). | Methods, pg. 6 |
| Number of Items | There was a total of 25 items, with the maximum number of items being 8 per page. | Methods, pg. 6 |
| Number of screens (pages) | 5 pages, and a progress bar was shown at the bottom of the page. | Methods, pg. 6 |
| Completeness check | All items (except for the screening questions at the start to determine eligibility) were voluntary and skippable. None of the items had "don't know/none of the above" options; questions on satisfaction had "neither" as an option. | Methods, pg. 6 |
| Review step | Participants had the option throughout the survey to use the "Back" and "Next" buttons to review answers. | Methods, pg. 6 |
| **Response rates** | | |
| Unique site visitor | Only participants or visitors completing at least the first page (agree to consent) and proceeding to the next page were counted. Thus, calculation of views or participation rates was not possible. | N/A |

| | | |
|---|---|---|
| View rate (Ratio of unique survey visitors/unique site visitors) | Only participants or visitors completing at least the first page (agree to consent) and proceeding to the next page were counted. Thus, calculation of views or participation rates was not possible. | N/A |
| Participation rate (Ratio of unique visitors who agreed to participate/unique first survey page visitors) | Only participants or visitors completing at least the first page (agree to consent) and proceeding to the next page were counted. Thus, calculation of views or participation rates was not possible. | N/A |
| Completion rate (Ratio of users who finished the survey/users who agreed to participate) | 296/397: 75% completion rate | Findings, pg. 10 |
| **Preventing multiple entries from the same individual** | | |
| Cookies used | Only one participation per device was possible using SurveyMonkey's multiple responses option which used cookies to assign a unique user identifier to each browser. | Methods, pg. 6 |
| IP check | IP addresses were not stored to ensure participant anonymity. | N/A |
| Log file analysis | Not used. | N/A |
| Registration | The survey was open, no registration was needed for entry to the survey. Participants accessed the survey by opening a link. | N/A |
| **Analysis** | | |
| Handling of incomplete questionnaires | Incomplete responses were analyzed if the mandatory eligibility questions were completed. Answers in the demographics question that were obviously false (e.g., weight/height incorrectly inputted) were excluded. | Methods, pg. 7 |
| Questionnaires submitted with an atypical timestamp | No respondents were removed from the survey for completing items too quickly | N/A |
| Statistical correction | No weighting scheme was used for the analysis of results. | N/A |

* Eysenbach G. Improving the quality of Web surveys: the Checklist for Reporting Results of Internet E-Surveys (CHERRIES). J Med Internet Res 2004;6:e34.
